# Small cuts, big questions: the impact of incision length in minimally invasive robotic cardiac surgery

**DOI:** 10.3389/fcvm.2025.1575779

**Published:** 2025-06-26

**Authors:** Thomas E. Rubino, Ariana Jackson, Martin Winter, Kristian Punu, Syed Faaz Ashraf, Keith Dufendach, Nicholas Hess, Rachel Deitz, Stephen D. Waterford, David Kaczorowski, Ibrahim Sultan, Johannes Bonatti

**Affiliations:** ^1^ Department of Cardiothoracic Surgery, University of Pittsburgh Medical Center, Pittsburgh, PA, United States; ^2^ Heart and Vascular Institute, University of Pittsburgh Medical Center, Pittsburgh, PA, United States

**Keywords:** cardiac surgery & percutaneous cardiovascular interventions, minimally invasive & robotic surgery, incision length, coronary artery surgery, clinical outcomes, valve surgery, TECAB, MIDCAB

## Abstract

**Introduction:**

Evidence on incision lengths for ports and cardiopulmonary bypass (CPB) cannulation in robotic cardiac surgery is limited. This study aimed to assess these metrics and influencing factors.

**Methods:**

204 patients underwent robotic mitral valve repair (MVR) (54.9%), totally endoscopic coronary artery bypass grafting (TECAB) (30.9%), and minimally invasive direct coronary artery bypass grafting (MIDCAB) (14.2%). Total incision length (TIL) was measured intraoperatively and defined as the sum of thoracic incisions, portholes, and incisions for cannulation. In both univariate and multivariate analyses, TIL was calculated based on demographic and intraoperative variables. Additionally, TIL was linked with postoperative outcomes.

**Results:**

The median length of thoracic access incisions and ports was 11.5 (5.0–51.0) cm, while for cannulation access, it was 5.0 (3.0–13.0) cm. The median total incision length was 16.5 (10.0–62.0) cm. Thirteen pre- and intraoperative variables were associated with TIL on univariate analysis. Multivariate analysis revealed that BMI (*p* = 0.003), procedure type (*p* < 0.001), conversion to sternotomy (*p* < 0.001), technical challenges (*p* = 0.034) and total procedure time (*p* < 0.001) were associated with extended incision length. Multivariate testing additionally showed an association of TIL with blood transfusion (*p* = 0.004) and hospital stay (*p* < 0.001).

**Conclusions:**

Incision length in robotic cardiac surgery is primarily linked to obesity, procedure type, surgical technical problems, conversion to sternotomy, and procedure time. Longer incisions are associated with an increased number of blood transfusions and longer hospital stay

## Introduction

Robotic technology enables cardiac surgery to be performed through small incisions and port holes rather than through a large sternotomy. Robotic cardiac surgery is mainly implicated in less invasive and totally endoscopic mitral valve surgery and coronary bypass grafting. Unless completely endoscopic, such as totally endoscopic coronary artery bypass grafting (TECAB) or totally endoscopic mitral valve repair, these procedures are carried out through a small working incision known as a minithoracotomy on the left or right chest to access the heart.

Existing literature describes the length of the minithoracotomy and robotic ports relatively non-quantitatively, often only referring to them in brief as “small”, or “short”. In those that do mention incision lengths, they are frequently described with a large range and are inconsistent across publications (1–12). In addition to these thoracic ports, the femoral artery and vein are also exposed in the groin through a small incision and the cannulae for cardiopulmonary bypass are inserted. Information on the access size for heart lung machine cannulation is also very limited (4).

Due to discrepancies and a lack of precisely measured evidence on this subject, this study aimed to quantify the lengths of individual incisions made on the patient's body, outline their locations, and identify factors influencing total incision length in robotically assisted coronary artery bypass and mitral valve surgery.

## Materials and methods

From July 2021 to April 2024, 218 patients underwent robotic cardiac surgery at UPMC Presbyterian or UPMC Passavant hospital. Of these, 112 (54.9%) were robotic mitral valve repair (MVR), 63 (30.9%) were robotic totally endoscopic coronary artery bypass grafting (TECAB), and 29 (14.2%) were robotically assisted minimally invasive direct coronary artery bypass grafting (MIDCAB). Of these 204 patients, the median age was 63 (24–85) years, 150 (73.5%) were male, and the median STS risk of mortality was 0.5 (0.1–4.5) %. The remaining 14 procedures (6.4%) were rare interventions, which were excluded from our analysis. Of the 204 patients included, nine (4.4%) underwent conversions to sternotomy due to bleeding issues (*n* = 5, 55.6%), complex mitral valve pathology requiring replacement (*n* = 1, 11.1%), the inability to tolerate single lung ventilation (*n* = 1, 11.1%), mitral valve re-repair due to access through minithoracotomy being restricted (*n* = 1, 11.1%), and placement of a vein graft to PCI target in hybrid coronary intervention for intraoperative ischemia (*n* = 1, 11.1%).

### Surgical technique

All procedures were carried out using the da Vinci Xi surgical robot [Intuitive, Sunnyvale, CA].

For robotic mitral valve repair, a minithoracotomy was placed in the 4th intercostal space on the right side of the chest. Three robotic ports were placed for the left and right arms of the robotic instrument and the left atrial retractor. Patients were cannulated in the right groin. An additional small incision was placed in the axillary fold for insertion of the left atrial suction tube and the Chitwood clamp. Patients were placed on cardiopulmonary bypass and cardioplegia was induced through a cardioplegia cannula in the aortic root after transthoracic aorta clamping. The left atrium was opened, and the mitral valve repair was performed with assistance by a patient side surgeon.

In robotic MIDCAB, instrument ports were placed in the 3rd, 5th, and 7th intercostal spaces on the patient's left lateral chest and docked to the surgical robot. CO2 was insufflated at pressures of 8 mmHg. The internal mammary artery was harvested in skeletonized technique and the pericardium was opened robotically. The target vessel was identified, and the location of the mini thoracotomy defined videoscopically. All patients were cannulated prophylactically in the groin or axillary depending on the grade of atherosclerosis on the aortoiliac level. After undocking the robot, a left sided mini thoracotomy was carried out and the graft to coronary artery anastomosis was performed on the beating heart using the Octopus Nuvo [Medtronic, Minneapolis, MN] for mechanical stabilization of the target vessel.

In robotic TECAB, the internal mammary harvesting was carried out as described for robotic MIDCAB. Additional ports were placed parasternally in the 4th intercostal interspace and subcostally for insertion of assisting instruments. Patients were cannulated in the groin using the Edwards Intraclude System [Edwards, Irvine, CA]. In procedures performed on the arrested heart, an endoaortic occlusion balloon was positioned into the aortic root under transesophageal echocardiography guidance. After initiation of cardiopulmonary bypass, the heart was arrested using the aortic occlusion balloon. In beating heart TECAB, the target vessel was stabilized using robotic long tip forceps brought in through a subcostal port and was snared with silastic tape. In both versions, the anastomosis was carried out in complete endoscopic fashion using robotic instrumentation.

In all three operations, patients were cannulated in the ipsilateral groin to the thoracic incisions. If more than mild aortoiliac atherosclerosis was present, we chose the axillary artery for arterial heart-lung machine access. All robotic MIDCAB patients were cannulated prophylactically, and a supportive pump run was initiated immediately in cases of technical difficulties or hemodynamic instability.

**Figure 1:**
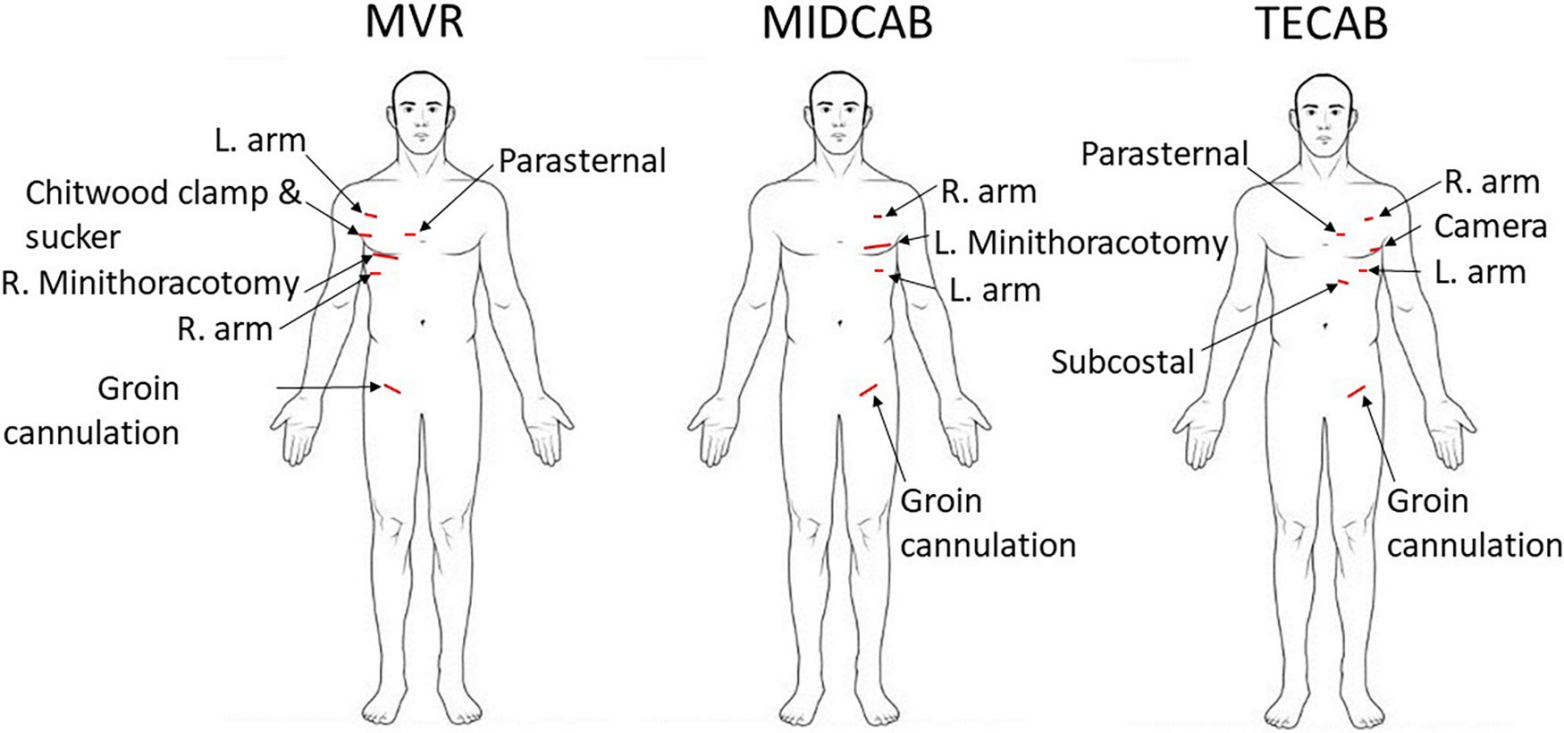
Schematic depicting access incisions for robotic mitral valve repair (MVR), robotic minimally invasive direct coronary artery bypass (MIDCAB), and robotic totally endoscopic coronary artery bypass (TECAB).

The incision lengths were measured intraoperatively at completion of the case with a sterile ruler. A scheme with the incision sites is shown in Figure 1. Total incision length (TIL) was defined as the sum of all thoracic ports and mini-incisions, the incisions for cannulation, and the length of the sternotomy if conversion to sternotomy became necessary.

### Ethical statement

This study was approved by the University of Pittsburgh Institutional Review Board (IRB #21080141) and patients gave informed consent.

### Statistical analysis

Statistical analysis was conducted using SPSS Statistics, version 28.0.1.1. Total incision length was correlated with preoperative, intraoperative, and postoperative variables. Categorical variables are shown as absolute values and percentages and continuous variables are shown as median, minimum, and maximum. Correlations were calculated using the Spearman's rho correlation coefficient. Differences between groups were calculated using Mann–Whitney *U*-test and Kruskall-Wallis test. A multivariate analysis for predictors of total incision length was conducted using multiple linear regression. Additionally, the association between total incision length and outcome variables was analyzed using multivariate analysis of variance (MANOVA) after converting the variable “total incision length” into quartiles. A *p*-value < 0.05 was considered statistically significant.

## Results

In this series of robotic cardiac surgical procedures, there was no hospital mortality, and no postoperative permanent stroke occurred. There were two revisions for bleeding, both done in a less invasive fashion, and one robotically in a TECAB patient. 121 out of 204 patients (59.3%) were extubated in the operating room. The median hospital stay was 4 (2–25) days.

### Incision length details

The various incision lengths for the three types of robotic operations are shown in Table 1. The median length of the port incisions ranged from 1 cm to 2 cm, with the overall range extending from 1 cm to 4 cm. The minithoracotomy in robotic MVR was shorter than the minithoracotomy in robotic MIDCAB [7.0 (5.0–15.0) cm vs. 9.0 (5.0–12.0 cm), *p* < 0.001]. The shortest thoracic incision length (sum of all chest incisions) was seen for robotic TECAB [7.5 (5.0–51.0) cm] as compared to MIDCAB [12.5 (7.0–17.0) cm] and MVR [12.0 (9.5–35.5) cm, *p* < 0.001]. The shortest total incision length was also seen in robotic TECAB with 12.5 [10.0–62.0] cm, (*p* = 0.022), which was 4.5 and 6.0 cm shorter than both MVR [17 (12.5–40.0) cm] and MIDCAB [18.5 (11.0–30.0) cm], respectively.

**Table 1:** Incision lengths by procedure type.

*n*	MVR	MIDCAB	TECAB	*p*-value	Total
	112	29	63		204
Left instrument port (cm)	1.0 [1.0–2.5]	1.0 [1.0–2.5]	1.0 [1.0–2.0]	0.488	1.0 [1.0–2.5]
Right instrument port (cm)	1.0 [1.0–3.0]	1.5 [1.0–2.5]	1.5 [1.0–2.5]	**<0** **.** **001**	1.0 [1.0–3.0]
Left atrial retractor port (cm)	1.0 [1.0–3.0]	n/a	n/a		
Chitwood clamp/Left atrial suction tube incision (cm)	2.0 [1.0–3.0]	n/a	n/a		
Octopus Nuvo incision (cm)	n/a	1.0 [1.0–1.5]	n/a		
Minithoracotomy (cm)	7.0 [5.0–15.0]	9.0 [5.0–12.0]	n/a	**<0** **.** **001**	7.0 [5.0–15.0]
Camera port (cm)	n/a	n/a	1.0 [1.0–3.0]		
Parasternal assistance port (cm)	n/a	n/a	1.0 [1.0–2.5]		
Subcostal port (cm)	n/a	n/a	2.0 [1.0–4.0]		
Thoracic incision length (cm)	12.0 [9.5–35.5]	12.5 [7.0–19.0]	7.5 [5.0–51.0]	**<0** **.** **001**	11.5 [5.0–51.0]
Cannulation incision length (cm)	5.0 [3.0–11.0]	5.0 [4.0–13.0]	5.0 [4.0–12.0]	**<0** **.** **001**	5.0 [3.0–13.0]
Sternotomy in converted patients (cm)	17.0 [17.0–22.0]	n/a	21.0 [15.0–22.0]	**<0** **.** **001**	21.0 [15.0–22.0]
Total incision length (cm)	17.0 [12.5–40.0]	18.5 [11.0–30.0]	12.5 (10.0–62.0)	**<0** **.** **001**	16.5 [10.0–62.0]

Values are measured in centimeters (cm) and represented with median and range.

Statistically significant results (*p* < 0.05) are highlighted in bold.

### Preoperative factors influencing incision length

**Figure 2:**
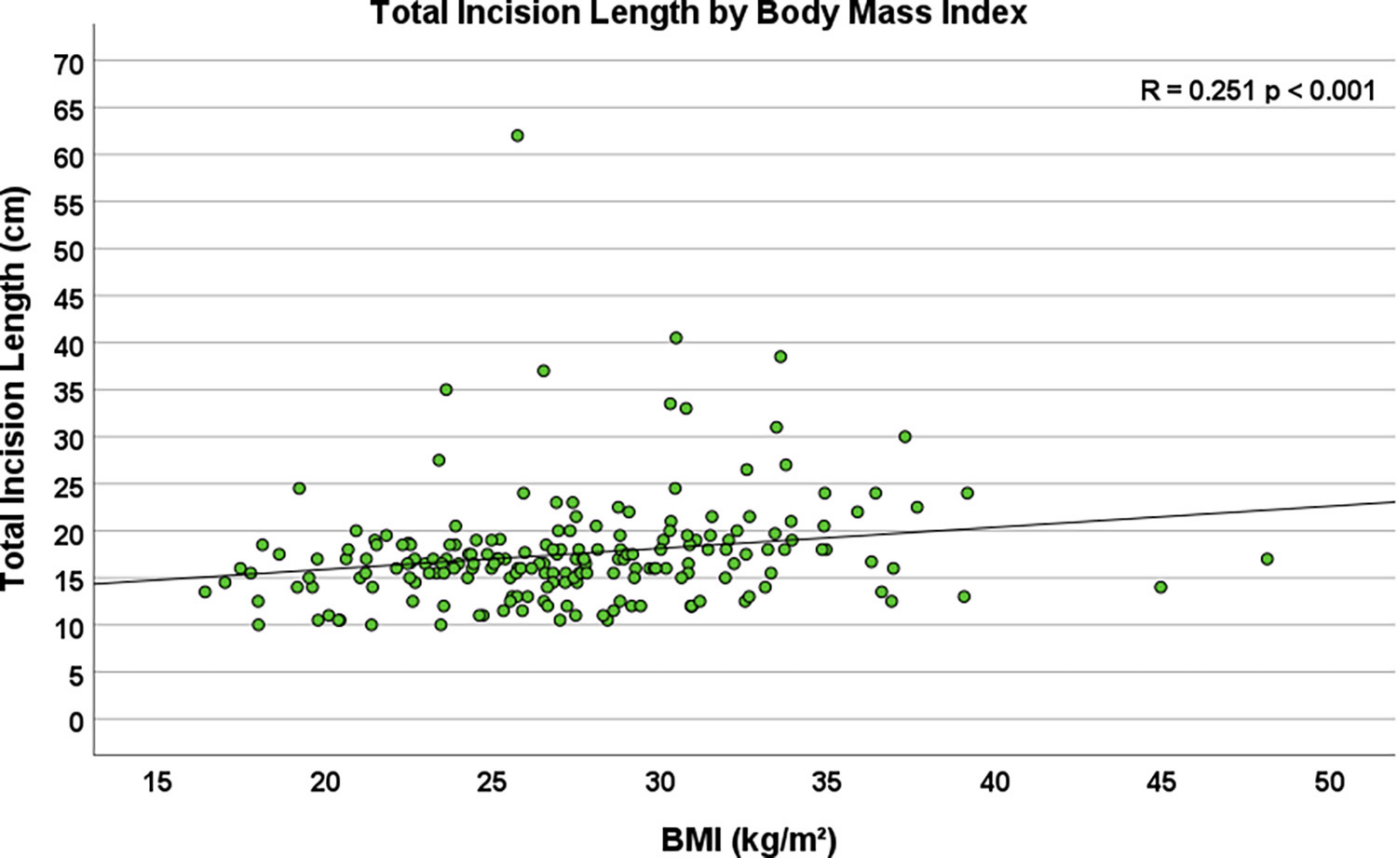
Scatterplot depicting the correlation between body mass Index (BMI) and total incision length (*n* = 204, R = 0.251, *p* < 0.001).

**Figure 3:**
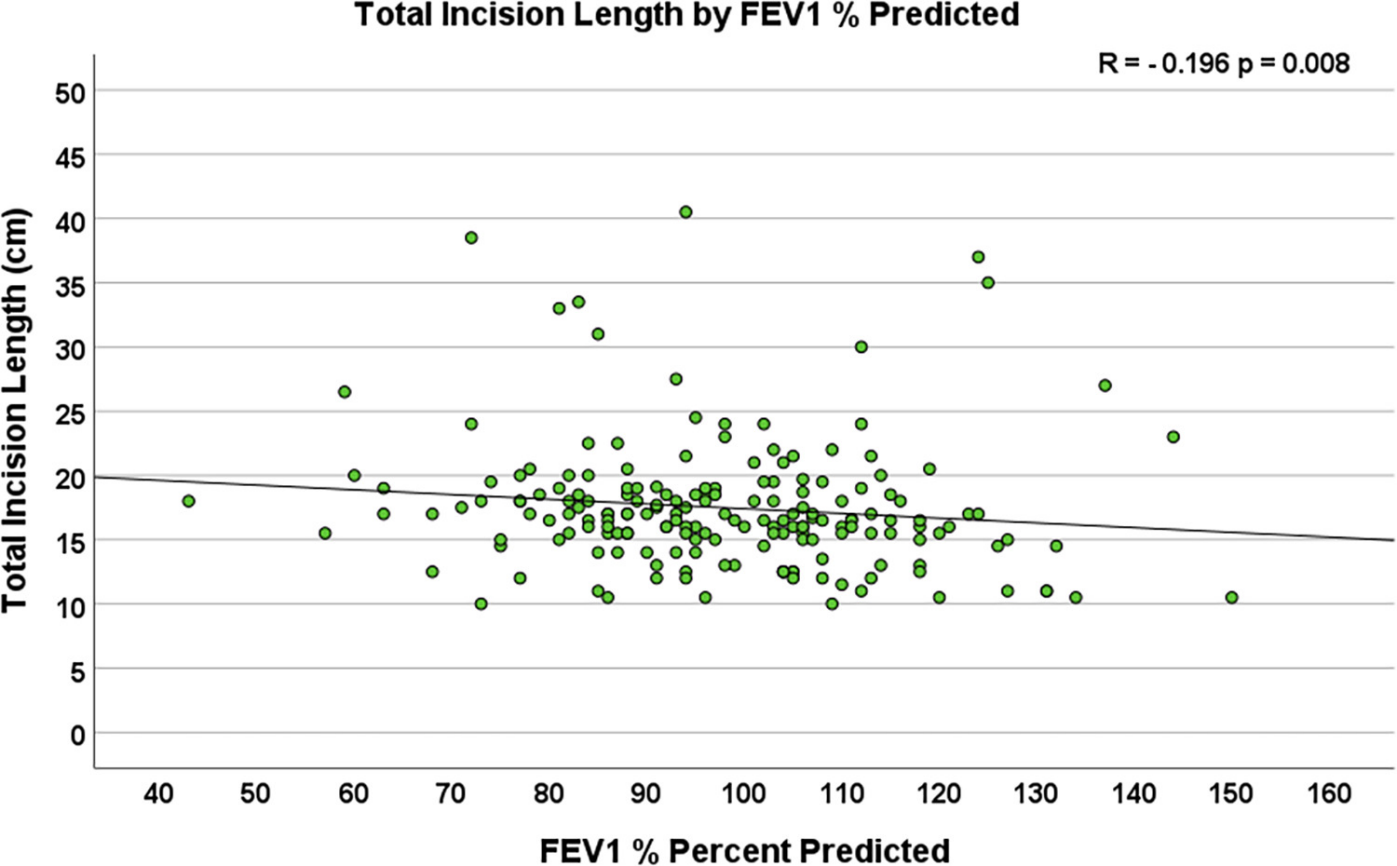
Scatterplot depicting the correlation between preoperative forced expiratory volume in One second (FEV1) % predicted and total incision length (*n* = 204, R = −0.196, *p* = 0.008).

Multiple body habitus measurements positively correlated with increased TIL, including weight (R = 0.179, *p* = 0.011), body surface area (R = 0.179, *p* = 0.011), and body mass index (R = 0.251, *p* < 0.001, Figure 2). Additionally, smoking status corresponded to longer total incision length (*p* = 0.039), and forced expiratory volume in one second (FEV1) (R = −0.196, *p* = 0.008, Figure 3) and diffusing capacity of the lungs for carbon monoxide (DLCO) (R = −0.173, *p* = 0.037) were inversely correlated with TIL. Table 2 lists the twenty-two preoperative variables investigated.

**Table 2:** Total incision length by demographics and preoperative variables.

Variable	Total incision length (cm)					
	*n* = 204	Percent (%)	Median	Range	R	*p*-value
Demographics						
Age (years)					−0.013	0.850
Sex						
Male	150	73.5	16.5	10.0–62.0		0.327
Female	54	26.5	17.0	10.0–38.5		
Height (cm)					−0.076	0.283
Weight (kg)					0.179	**0** **.** **011**
BMI (kg/m^2^)					0.251	**<0** **.** **001**
BSA (m^2^)					0.179	**0** **.** **011**
LVEF (%)					0.054	0.451
Risk factors						
Smoking						
No	120	58.8	16.0	10.5–62.0		**0** **.** **039**
Former	60	29.4	17.5	10.0–40.5		
Current	24	11.8	16.5	10.0–30.0		
Diabetes mellitus						
No	167	81.9	16.5	10.0–62.0		0.096
Yes	37	18.1	17.5	10.0–38.5		
Last A1C level					0.023	0.746
Hypertension						
No	53	26.0	17.0	10.5–35.0		0.606
Yes	151	74.0	16.5	10.0–62.0		
Comorbidities						
COPD						
No	156	76.5	16.5	10.0–62.0		0.138
Yes	48	23.5	17.0	12.0–38.5		
FEV1% predicted					−0.196	**0** **.** **008**
DLCO % predicted					−0.173	**0** **.** **037**
Creatinine					−0.084	0.241
CVD						
No	184	90.2	16.5	10.0–62.0		0.616
Yes	20	9.8	17.0	10.0–20.0		
PVD						
No	199	97.5	16.5	10.0–62.0		0.426
Yes	5	2.5	18.0	15.5–20.0		
Liver disease						
No	199	97.5	16.5	10.0–62.0		0.826
Yes	5	2.5	14.0	12.0–33.5		
Preoperative labs						
WBC					−0.028	0.700
Hemoglobin					−0.002	0.981
Hematocrit					0.023	0.750
Platelet count					0.007	0.926
Total albumin					−0.067	0.349

Values are measured in centimeters (cm) and represented with median and range.

Statistically significant results (*p* < 0.05) are highlighted in bold.

### Intraoperative factors influencing incision length

Incisions were longer in patients with complex operations [17.0 (11.0–40.0) cm] defined as procedures technically more extensive than placement of a left internal mammary artery (LIMA) to the left anterior descending artery (LAD) or correction of a simple P2 pathology. Patients who underwent the less complex procedure had a TIL of 16.5 [10.0–62.0] cm, (*p* = 0.012). In patients where no surgical technical problem occurred, the total incision length median was 16.0 (10.0–24) cm. In those patients where technical challenges did occur, the incision length median was 17.0 (10.0–62.0) cm. The total incision length for patients converted to sternotomy was 35.0 (27.5–62.0) cm as compared to 16.5 (10.0–30.0) cm those who did not require conversion (*p* < 0.001).

**Figure 4:**
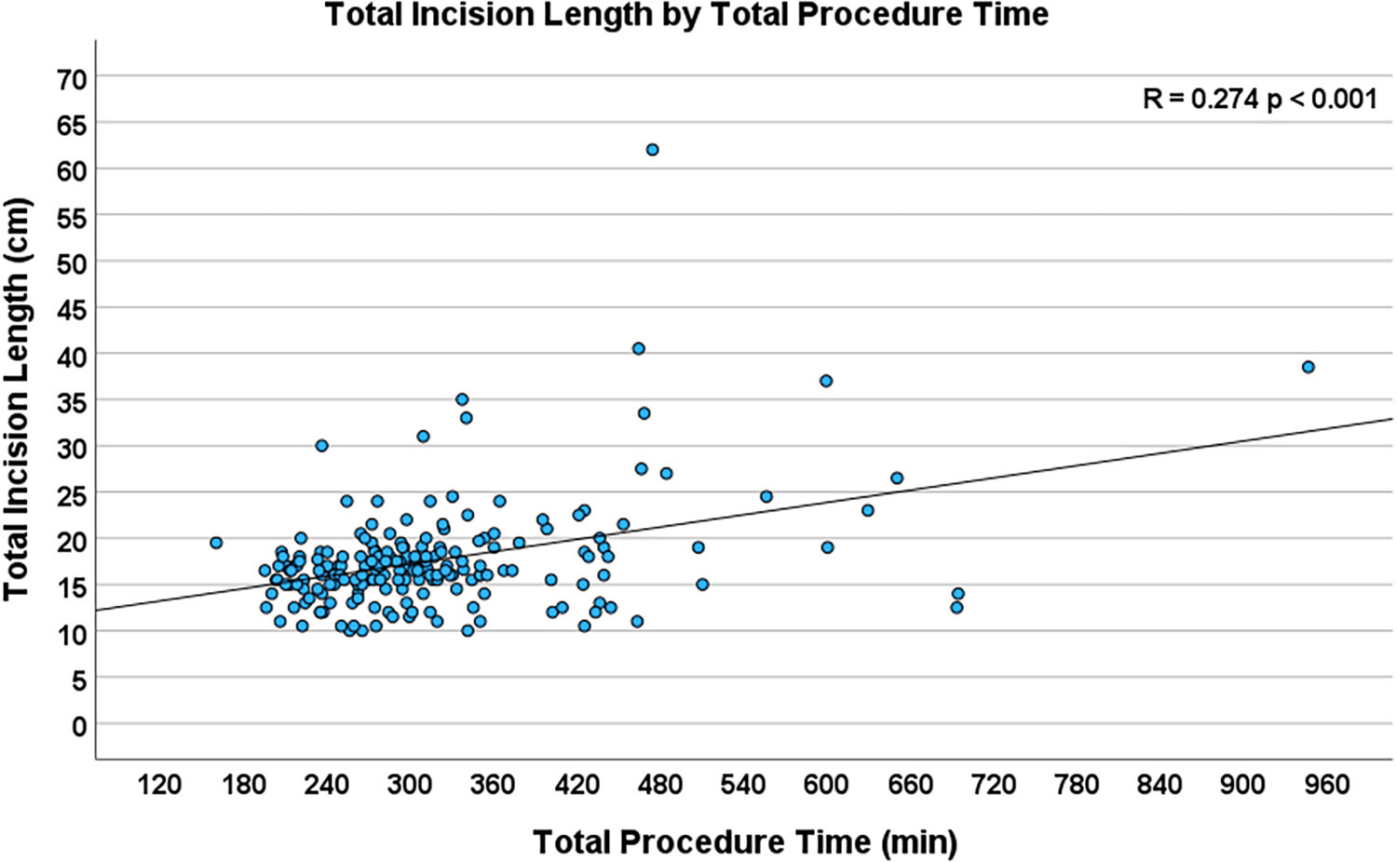
Scatterplot depicting the correlation between total procedure time and total incision length (*n* = 204, R = 0.274, *p* < 0.001).

Operative times, which included cardiopulmonary bypass time (R = 0.224, *p* = 0.001), myocardial ischemic time (R = 0.208, *p* = 0.003), and total procedure time (R = 0.274, *p* < 0.001, Figure 4), were highly significantly associated with total incision length. Extubation status in the operating room did not have a significant association with TIL. Table 3 lists the seven intraoperative and eleven postoperative variables investigated.

**Table 3:** Total incision length by intra-and postoperative variables.

Variable	Total incision length (cm)					
	*n* = 204	Percent (%)	Median	Range	R	*p*-value
Intraoperative variables						
Conversion to sternotomy						
No	195	95.6	16.5	10.0–30.0		**<0.001**
Yes	9	4.4	35.0	27.5–62.0		
Complex procedure						
No	133	65.2	16.5	10.0–62.0		**0.012**
Yes	71	34.8	17.0	11.0–40.0		
Any technical challenge						
No	84	41.2	16.0	10.0–24.0		**0.044**
Yes	120	58.8	17.0	10.0–62.0		
Cannulation problem						
No	164	80.4	16.5	10.0–40.5		0.784
Yes	40	19.6	16.5	10.0–62.0		
CPB time					0.224	**0.001**
Myocardial ischemic time					0.208	**0.003**
Total procedure time					0.274	**<0.001**
Postoperative variables						
Ventilation time					0.197	**0.005**
Total number of RBC units transfused					0.198	**0.005**
Total days in ICU					0.124	0.083
Total days in hospital					0.229	**0.001**
No	202		16.5	10.0–62.0		0.555
Yes	2		18.0	16.5–19.0		
Postop permenant neurologic deficit						
No						n/a
Yes						
Postop new atrial fibrilation						
No	153	75.0	16.5	10.0–62.0		0.596
Yes	51	25.0	17.0	10.5–33.5		
Postop pnuemonia						
No						n/a
Yes						
Postop renal failure						
No						n/a
Yes						
Postop dialysis						
No						n/a
Yes						
Postop deep thoracic wound infection						
No						n/a
Yes						

Values are measured in centimeters (cm) and represented with median and range.

Statistically significant results (*p* < 0.05) are highlighted in bold.

**Figure 5:**
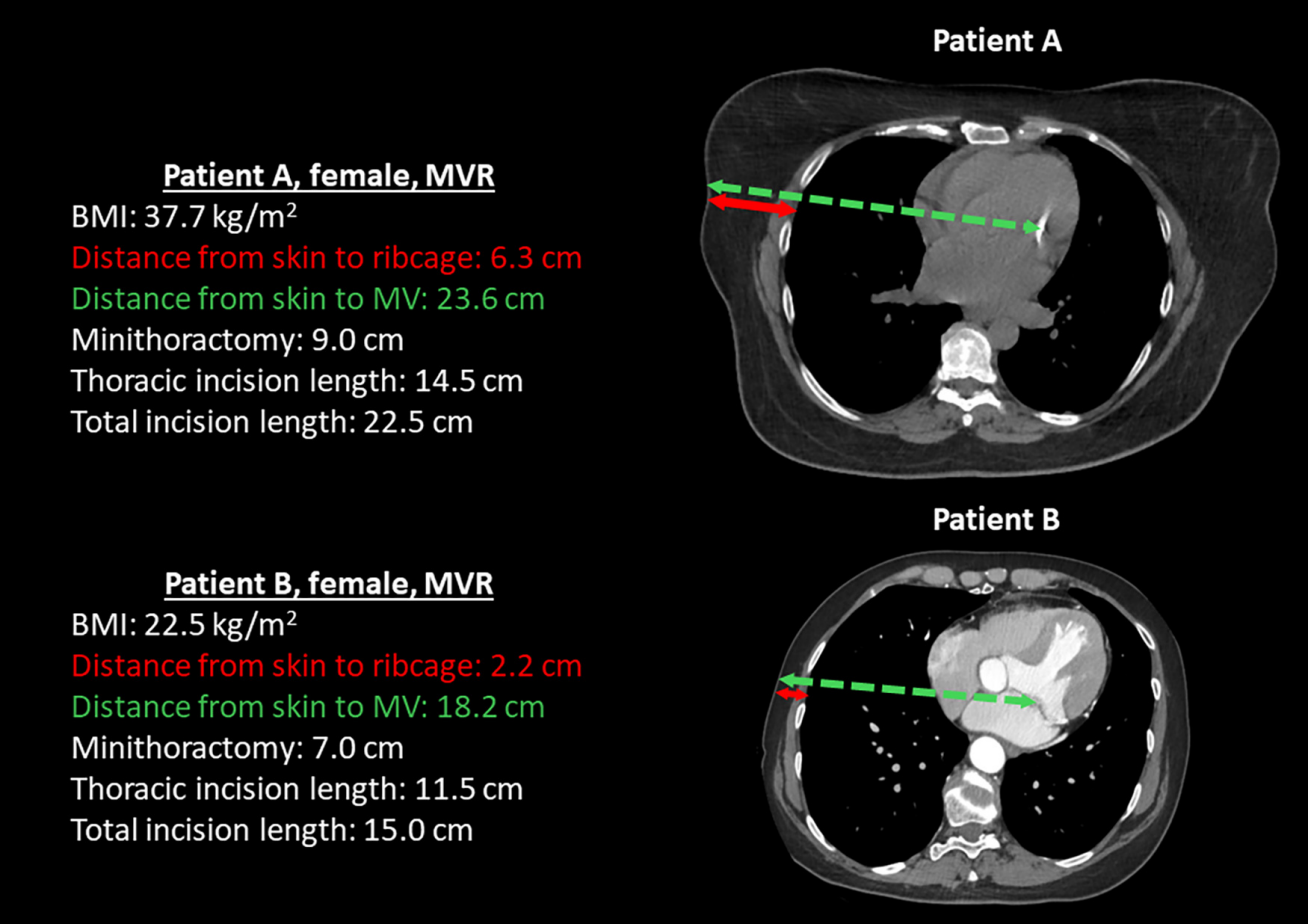
Representative CT scans of two female patients who underwent mitral valve repair (MVR), illustrating anatomical differences between an obese patient (patient A) and a non-obese patient (patient B). The scans highlight variations in soft tissue thickness, the distance to the mitral valve, and the corresponding incision lengths required for surgical access.

Table 4 shows the univariate association of pre- and intraoperative factors with TIL and the correlation of TIL with postoperative outcomes for each of the three operations studied. BMI was highly significantly associated with TIL in both robotic mitral valve repair and robotic TECAB and there was a corresponding trend in robotic MIDCAB. Increased tissue thickness in obese patients requires larger access to reach the ribcage and mitral valve. Figure 5 shows representative CT scans that demonstrate the anatomical differences between an obese and a non-obese patient, highlighting variations in soft tissue thickness, distance to the mitral valve, and their corresponding incision lengths. Conversion to sternotomy also showed a relation with increased incision length in robotic MVR and TECAB. Of note no conversion happened in robotic MIDCAB. Total procedure time was significantly associated with TIL in all three procedure variations.

**Table 4:** Univariate analysis of pre- and intraoperative factors and postoperative outcomes with total incision length (TIL) for each of the three operations studied: robotic mitral valve repair (MVR), robotic minimally invasive direct coronary artery bypass grafting (MIDCAB), and totally endoscopic coronary artery bypass grafting (TECAB).

*n* = 204	Robotic MVR				Robotic MIDCAB				Robotic TECAB			
	112				29				63			
	Median	Range	R	*p*-value	Median	Range	R	*p*-value	Median	Range	R	*p*-value
Preop and intraop factors and TIL												
Weight			0.274	**0** **.** **003**			0.367	0.054			0.272	**0** **.** **035**
BMI			0.392	**<0** **.** **001**			0.368	0.054			0.342	**0** **.** **008**
BSA			0.274	**0** **.** **003**			0.367	0.054			0.272	**0** **.** **035**
Smoking				**0** **.** **004**				0.266				0.787
No	17.0	13.5–26.5			16.0	13.0–24.0			13.0	10.5–62.0		
Current	16.0	12.5–17.0			19.0	11.0–30.0			12.5	10.0–24.0		
Former	18.5	15.0–40.5			18.5	16.0–27.0			12.5	10.0–33.0		
FEV1% predicted			−0.84	0.392			−0.054	0.589			−0.252	0.074
DLCO % predicted			−0.105	0.343			−0.054	0.807			−0.228	0.467
Conversion to sternotomy				**<0** **.** **001**				n/a				**0** **.** **015**
No	17.0	12.5–26.5							12.5	10.0–24.0		
Yes	37.0	33.5–40.5							33.0	27.5–62.0		
Complex procedure				0.106				0.643				0.158
No	17.0	12.5–35.0			18.0	11.0–30.0			12.5	10.0–62.0		
Yes	17.0	13.5–40.5			19.0	16.0–20.0			14.0	11.0–37.0		
Any technical challenge				**0** **.** **006**				0.118				0.282
No	16.5	12.5–24.0			17.5	11.0–21.0			12.5	10.0–20.0		
Yes	17.6	14.5–40.5			19.0	13.0–30.0			13.5	10.0–62.0		
CPB time			0.298	**0** **.** **001**			0.427	**0** **.** **024**			0.137	0.297
Myocardial Ischemic time			0.271	**0** **.** **004**			0.303	0.132			0.101	0.450
Total procedure time			0.471	**<0** **.** **001**			0.548	**0** **.** **003**			0.283	**0** **.** **033**
Postop outcomes and TIL												
Ventilation time			0.320	**<0** **.** **001**			0.208	0.287			0.289	**0** **.** **028**
Number of blood Transfusions			0.188	**0** **.** **048**			0.209	0.285			0.287	**0** **.** **026**
Hospital stay length			0.270	**0** **.** **004**			0.040	0.839			0.380	**0** **.** **003**

Values are measured in centimeters (cm) and represented with median and range.

Statistically significant results (*p* < 0.05) are highlighted in bold.

### Incision length and postoperative outcomes

**Figure 6:**
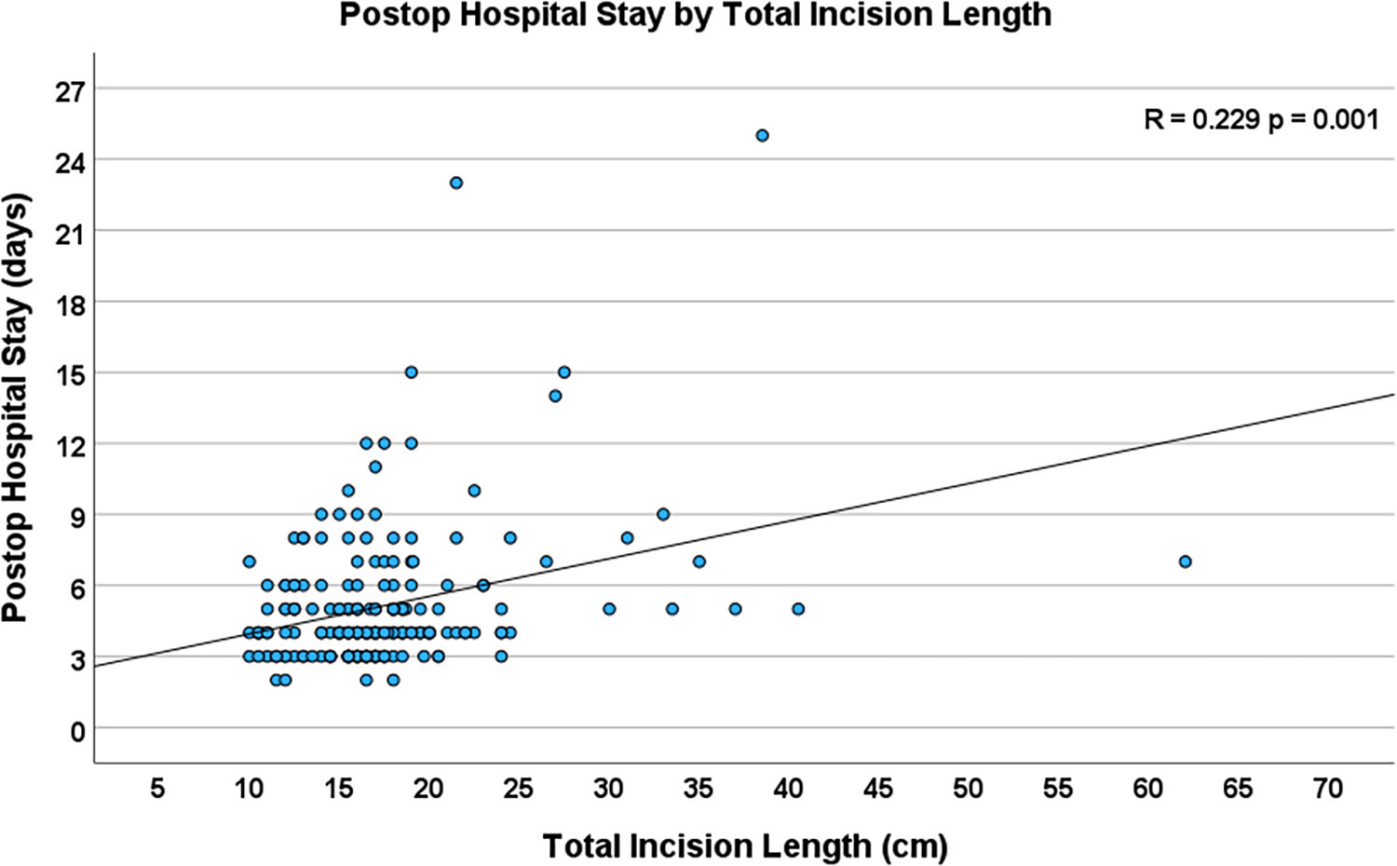
Scatterplot depicting the correlation between total incision length and total postoperative hospital stay (*n* = 204, R = 0.229, *p* = 0.001).

Increased total incision length was significantly associated with increased postoperative hospital length of stay (R = 0.229, *p* = 0.001, Figure 6). Additionally, longer incision lengths correlated with increased ventilation time (R = 0.197, *p* = 0.005), and an increased number of units of blood transfusions (R = 0.227, *p* = 0.019). The total incision length was 17.0 (10.5–40.5) cm in the patients who had reached full activity at the 4 weeks postoperative visit and 16.5 (10.0–62.0) cm in those who had not reached full activity level at the same time point (*p* = 0.153).

Table 5 presents both the univariate and multivariate predictors of TIL, along with the univariate and multivariate associations between TIL and postoperative outcome variables. It also includes a sub analysis of patients who did not undergo conversion to sternotomy.

**Table 5:** Univariate and multivariate analysis of predictors of total incision length (TIL) as well as the univariate and multivariate associations between TIL and postoperative outcome variables.

Variable	Univariate Analysis	Multivariate Analysis
	*p*-value	*p*-value
Predictors of total incision length (TIL)		
Demographics		
Weight (kg)	**0**.**011**	0.433
BMI (kg/m^2^)	**<0** **.** **001**	**0**.**003**
BSA (m^2^)	**0**.**011**	0.376
Risk factors		
Smoking	**0**.**039**	0.466
FEV1% predicted	**0**.**008**	0.517
DLCO % predicted	**0**.**037**	0.898
Intraoperative variables		
Procedure type	**<0** **.** **001**	**<0** **.** **001**
Complex procedure	**0**.**012**	0.648
Any technical challenge	**0**.**044**	**0**.**034**
Conversion to sternotomy	**<0** **.** **001**	**<0** **.** **001**
CPB time	**0**.**001**	0.856
Myocardial ischemic time	**0**.**003**	0.099
Total procedure time	**<0** **.** **001**	**<0** **.** **001**
TIL association with postoperative outcome		
Postoperative variables		
Ventilation time	**0**.**005**	0.089
Number of blood transfusions	**0**.**019**	**0**.**004**
Hospital stay	**0**.**001**	**<0**.**001**

Multivariate predictors remained statistically significant even when patients who underwent conversion to sternotomy were excluded.

Statistically significant results (*p* < 0.05) are highlighted in bold.

## Discussion

### Preoperative variables

Before conducting this retrospective study, we hypothesized that anatomical factors, such as body habitus, would strongly influence the length of these incisions. Our work suggests correlations between BMI and total incision length in robotic cardiac surgery. This finding was observed by Brunaud et al., where they identified a positive correlation between incision length and body mass index in standard thyroidectomy and parathyroidectomy procedures (13). While our literature search did not reveal corresponding studies in cardiac surgery publications, we found multiple articles describing how BMI influenced postoperative outcomes, including increased mortality, morbidity, and cost (14, 15). Notably, one smaller study found that robotic mitral valve surgery was no less safe to perform in patients with a BMI over 30 compared to those with a BMI under 30 (16). While there seems to be some conflicting evidence, the literature generally leans towards a higher BMI negatively influencing outcomes, with our study suggesting longer incisions and increased tissue trauma in this patient population. We also found that total incision length correlated positively with weight and body surface area. Additionally, on univariate analysis, we noted a longer TIL in patients with impaired lung function, namely lower FEV1 (% of predicted) and DLCO. We could not identify any papers in the literature elaborating on this finding.

### Intraoperative variables

The final lengths of the port incisions were found to range between 1 and 3 cm, despite the inserted metal robotic ports being 0.8 cm in diameter. Possible explanations for this include the surgeon initially making a slightly longer incision, the movement of the metal port extending the incision during procedures requiring greater arm mobility, and enlarging the port incision due to bleeding from the port. The literature does not specifically expand on this question, but the reported estimates for port sizes are in a similar range of our measurements (9–12).

It was interesting for us to observe that the right instrument port in MIDCAB and TECAB was significantly longer than in robotic MVR. A potential explanation is that this port travels through the pectoralis major muscle, which is well vascularized and tends to bleed, and occasionally port enlargements are necessary. In addition, the excursions of the right instrument in robotic coronary bypass surgery are more extensive than in robotic mitral surgery in which the workspace is relatively confined.

Additionally, the MIDCAB minithoracotomy was, on average, 2 cm longer than the corresponding minithoracotomy for robotic MVR. We believe that this occurs because in robotic MVR, the surgeon operates within a very narrow, cone-shaped operative field that has little variability between patients, whereas in robotic MIDCAB, the anatomical location of a patient's target vessels can be considerably variable.

It was anticipated to find variations in incision length among the three operations studied. TECAB, performed entirely endoscopically through ports, showed the shortest total incision length. In contrast, both MIDCAB and robotic MVR, which involve a minithoracotomy, resulted in similar, longer incisions.

Early and late publications on robotic mitral valve surgery do not present precise lengths of incisions. For example, the primary right minithoracotomy in MVR has been reported from 3 to 8 cm in length (1–5). A 3–6 cm incision is similarly characterized for the left minithoracotomy in MIDCAB (6, 7). Cannulation incisions are also infrequently discussed. In one of the first published series of mitral valve repair, Nifong states a 5–6 cm minithoracotomy in robotic MVR, and reports a 2 cm groin incision for cannulation (4).

Incisions in robotic mitral valve repair are likely slightly longer than those in classic videoscopic non-robotic mitral valve repair because, in the latter, the surgeon uses only one port for the camera in addition to the minithoracotomy. In robotic procedures, three additional ports are placed: one for the right instrument, one for the left instrument, and one for the left atrial retractor. We believe that the added level of precision in robotic approaches justifies these extra port incisions. In our literature search for this study, we did not find precise measurements for the robotic port incisions in robotic TECAB. Balkhy et al. describes placement of “standard” ports in the 2nd, 4th, and 6th intercostal spaces, which on the robotic system he used corresponds to three 8 mm incisions. In addition, he inserted a 12 mm subcostal port and a 15 mm port in the 2nd intercostal space for insertion the Flex A automated anastomotic device (10). Yang et al. in a series of robotic coronary bypass surgery reports three 0.8–1 cm incisions for the camera and two instrument ports (12). Overall, for robotic ports, which TECAB uses exclusively, publications describe the diameters as ranging from 0.5 to 2 cm (8–12).

One of our key findings was that as operation times increased, the total incision length also increased. Lee et al. investigated this relationship in laparoscopic vs. single-incision cholecystectomy and, like us, found that longer incisions were associated with significantly longer operating times (17). Similarly, the research by Brunaud and colleagues on thyroid and parathyroid resection considered the relationship of operation time and incision length and found a significant correlation with the duration of the procedure (13). On the other hand, Chung et al. studied traditional vs. mini-incision total hip replacement and found some similar outcomes for the shorter incision operation, such as a shorter length of hospital stay, but they did not state that longer incisions were associated with significantly longer operating times (18).

Brunaud's group attributed increases in incision lengths to the surgical complexity and case-specific technical difficulties (13). Similarly, we observed a slight increase in total incision length (TIL) in patients who underwent complex robotic coronary bypass surgery or mitral valve repair. Our study also indicated that longer operating times are more likely to occur when technical challenges are encountered. These challenges may require additional incisions to be made, or previously made incisions might need to be lengthened to achieve adequate vision and space for addressing the issues. These problems varied in complexity, with the most common consisting of port placement and cannulation problems, bleeding issues, unforeseen anatomical variations, intraoperative revisions of primary reconstructions e.g., for residual mitral valve regurgitation, and problems related to robotic technology. Technical challenges in surgery, even when minor, were meticulously documented in our operative reports and were present in 41% of operations. Technical challenges appeared to lead to an increase in TIL of 1 cm, which seems acceptable.

In 4.4% of patients in our series, technical challenges required the conversion of the robotic surgical approach to a traditional, midline sternotomy for patient safety and the assurance of a successful coronary revascularization or mitral valve repair. There were no conversions in our MIDCAB cases. Our findings were consistent with early studies, such as one by Glower et al., which compared incision lengths in sternotomy and port access mitral repair and found them to measure 26 cm and 8 cm, respectively (19). In comparison, the sternotomies in our conversions ranged from 15 cm to 22 cm. When placing our overall incision length of 16.5 cm in robotic cardiac surgery against the average sternotomy of 21 cm, it can be noted that robotic coronary and mitral surgery reduced the incision length by approximately 22% in our series. Although this reduction might seem less significant than anticipated, it is important to consider that the smaller incisions distribute the surgical trauma more evenly and leave the breastbone completely intact. This also does not account for the additional large incision necessary to harvest the saphenous vein commonly used in open coronary artery bypass grafting operations.

### Are the predictors for TIL individually present in robotic MVR, MIDCAB, and TECAB?

The primary goal of our study was to examine robotic cardiac surgery as a whole, encompassing various procedures. A sub-analysis revealed that the univariate predictors, conversion to sternotomy and procedure time, were associated with TIL in all procedure subtypes analyzed. Multivariate testing confirmed their association with TIL, and in our opinion, both factors are plausible.

### Incision length and perioperative outcomes

In a paper published in 2011, Bonatti et al. demonstrated a strong correlation between procedure time in robotic TECAB and the number of blood transfusions (20). This agrees with our finding that the number of transfusions was related to incision length and surgical trauma.

As can be estimated from our graphs, the hospital stay was approximately 5 days if the incision length was roughly between 10 cm and 15 cm. If the incision length was between 35 and 40 cm, the hospital stay was around 7 days. Multiple earlier studies comparing smaller incisions in cardiac surgery with sternotomy have demonstrated that reduced incision length resulted in shorter hospital stay (1, 19–23). Other papers, however, could not show such an effect (24, 25).

### Multivariate analysis of factors

Among the preoperative factors associated with increased incision length, only obesity remained significant on multiple regression analysis. This finding suggests that patients with a higher BMI may require closer attention and preparation for potential increased surgical trauma. Procedure type was consistently a strong predictor of total incision length in multivariate testing, highlighting the need to develop fully endoscopic procedures, which resulted in the smallest TILs. Additionally, increased surgical trauma due to technical difficulties and the need for full chest opening via sternotomy were strongly associated with increased incision length in multivariate analysis. Continued refinement of procedural techniques and training methods is likely necessary to address this challenge.

Total procedure time was also associated with longer incisions in multivariate testing, suggesting that more extended surgical work was needed for these patients. The analysis revealed a correlation between longer TIL and an increased number of blood transfusions and longer hospital stays, indicating that minimizing incisional trauma could reduce postoperative morbidity in robotic cardiac surgery.

Even when patients who were converted to sternotomy were excluded, the majority of the factors we analyzed remained statistically significant. This confirms that conversion cases did not significantly skew the data, ensuring consistency in patients who completed a robotic operative course via ports or mini-incisions.

### Sequelae of increased surgical incision length in experimental models

The decreased clinical trauma we observed is supported by laboratory studies that found increased surgical trauma correlated with elevated levels of inflammatory markers and activation of immune cells (26). Ioannidis et al. focused on incision length in a mouse model. They found that serum inflammatory markers, including CINC1/IL-8, TNF-α, and NO, significantly increased in mice who received incisions vs. the control group who did not have an incision. Additionally, the serum inflammatory markers increased proportionally for mice who received 10 cm incisions compared to the group who received a 1 cm incision, ultimately showing that there is a greater systemic inflammatory response that correlates with incision length (27).

### Future directions

We hope that future papers report precise measurements of incision length and explore it as a variable of significance, given its importance for patient cosmesis and recovery. Furthermore, it would be desirable to further reduce the incision length in robotically assisted, minimally invasive cardiac surgery. One strategy would be to perform more totally endoscopic procedures such as the totally endoscopic approach for mitral valve repair which has been developed by Dr. Sloane Guy and colleagues. They used five 8 mm ports instead of a right minithoracotomy for robotic mitral valve repair (21). As our program was recently created, we have not yet begun performing this version of mitral valve repair but plan to do so in the future. Another strategy is to use percutaneous heart-lung machine cannulation and closure devices to eliminate the need for groin incisions. This method has also been carried out successfully in the clinical setting (22, 23). Reducing the size of robotic ports will also depend on the efforts of the manufacturers of robotic devices. There are, however, challenges in reducing the port and instrument diameters as the function and strengths of the robotic instruments may be impaired. It will be up to the surgeon and their team to consciously reduce the size of the incisions to the smallest possible length while respecting all aspects of patient safety and procedure efficacy. As any robotic program matures, a reduction in technical difficulties and conversions to sternotomy can be expected. Although there is an obvious difference in incision length between approaches, it may be reasonable to advocate for incision length to become a mandatory metric in studies comparing minimally invasive and conventional cardiac surgery. Based on our findings, greater awareness of incision length—both within surgical teams and the broader cardiac surgery community—would be beneficial. One potential direction for future work is to compare the preoperatively marked incision length with the final intraoperative length achieved. Incorporating these metrics into robotic cardiac surgery databases would help standardize reporting and support more meaningful comparisons across techniques.

### Limitations

Our study is a small, single-center, single-surgeon, retrospective analysis without a control group. Low risk and anatomically well-suited patients were primarily selected for the robotic approach. Although the primary surgeon was experienced in robotic cardiac surgery, a new robotic cardiac surgery program was installed and the associated team learning effects were present.

## Conclusions

We conclude that the primary incisions for robotic cardiac surgery are relatively small, but additional access for robotic ports and cannulation lead to a considerable total incision length. However, total incision length is still markedly shorter than a standard sternotomy, the surgical trauma is dispersed, and the sternum remains intact. Total incision length is strongly dependent on procedure type, with the totally endoscopic approach exhibiting the shortest overall measurement. Incisions are generally longer in obese patients. Technical difficulties, conversions to sternotomy, and total procedure time are strongly correlated with total incision length. According to our data, longer incisions are associated with increased need for blood transfusions and longer hospital stay. These results remain consistent even when patients who were converted to sternotomy are excluded from analysis.

## Data Availability

The raw data supporting the conclusions of this article will be made available by the authors, without undue reservation.
